# Safety and Efficiency of Various Pancreatic Enucleation Procedures: A Systematic Review and Meta-Analysis

**DOI:** 10.3390/jcm15093543

**Published:** 2026-05-06

**Authors:** Deqiang Zhou, Feng Tan, Zihe Wang, Ning Xia, Xing Huang, Li Wang, Shijie Cai, Bole Tian, Junjie Xiong

**Affiliations:** 1 Division of Pancreatic Surgery, Department of General Surgery, West China Hospital, Sichuan University, No. 37 Guoxue Alley, Chengdu 610041, China; zhoudq2002@126.com (D.Z.); tfjjg520@126.com (F.T.); clab0709@ox.ac.uk (Z.W.); xianing@stu.scu.edu.cn (N.X.); huangxing@wchscu.cn (X.H.); neo_wl@163.com (L.W.); hxtbl0338@163.com (B.T.); 2 Nuffield Division of Clinical Laboratory Sciences, Radcliffe Department of Medicine, John Radcliffe Hospital, University of Oxford, Oxford OX1 3QR, UK; shijie.cai@ndcls.ox.ac.uk

**Keywords:** pancreatic enucleation, minimally invasive surgery, laparoscopic surgery, robotic surgery, pancreatic tumor, wound infection, blood loss, meta-analysis

## Abstract

**Objective**: This study aimed to systematically compare the short-term outcomes of minimally invasive pancreatic enucleation (MI-pEn), including laparoscopic and robotic-assisted approaches, and open pancreatic enucleation (O-pEn). **Methods**: A systematic search of PubMed, MEDLINE, Embase, and Web of Science was conducted for studies published between January 1990 and December 2025 that compared various types of pancreatic enucleation. The literature screening, data extraction, and quality assessment followed the PRISMA guidelines. The meta-analysis was performed using RevMan 5.4.1 and R 4.3.0. **Results**: Fifteen studies were included, with thirteen comparative studies (463 MI-pEn, 547 O-pEn) incorporated into the meta-analysis. Two studies comparing laparoscopic and robot-assisted enucleation were also included. No significant difference in clinically relevant postoperative pancreatic fistula (CR-POPF) was detected between MI-pEn and O-pEn (OR = 0.78; 95% CI: 0.56–1.07; *p* = 0.12). However, MI-pEn was associated with significantly reduced operation time (MD = −21.24; *p* = 0.01), blood loss (MD = −75.88; *p* < 0.00001), hospital stay (MD = −2.07; *p* = 0.001), and wound infection (OR = 0.3; *p* = 0.03). Direct comparisons between robotic and laparoscopic enucleation revealed no significant differences in any outcomes. **Conclusions**: MI-pEn is safe and feasible and offers advantages in terms of operative time, blood loss, and recovery without increasing complications. Robotic and laparoscopic approaches yield comparable short-term outcomes in pancreatic enucleation, although the potential advantage of robotic surgery in reducing pancreatic fistula risk warrants further validation.

## 1. Introduction

Pancreatic enucleation is an established parenchyma-sparing technique for benign or low-grade malignant pancreatic tumors and offers the dual benefit of complete tumor resection and maximal preservation of pancreatic function [1,2]. While this approach avoids the significant long-term metabolic morbidity associated with major resections such as regular pancreatectomy, it is technically demanding [3]. The primary challenge remains the precise intraoperative identification of tumor margins and the protection of the main pancreatic duct (MPD) [4], with postoperative pancreatic fistula rates reported to be between 30% and 40% [5]. Indications for pancreatic enucleation include benign or low-grade malignant tumors such as insulinomas, solid pseudopapillary neoplasms, and G1/G2 neuroendocrine tumors. The decision is typically made by a multidisciplinary team, and enucleation is considered an alternative to formal pancreatectomy when the tumor is superficial and at a safe distance from the main pancreatic duct.

In recent years, the principles of minimally invasive surgery have been progressively applied to pancreatic surgery. Laparoscopic and robotic platforms, enhanced by advanced imaging modalities such as intraoperative ultrasound and fluorescence navigation, have the potential to overcome some of the technical hurdles of enucleation by offering superior visualization and precision [6,7]. Consequently, the application of minimally invasive pancreatic enucleation (MI-pEn) is expanding, driven by the promise of reduced trauma and faster recovery [7].

Despite this growing interest, the widespread adoption of MI-pEn is not without significant debate and ongoing challenges [8]. Recent large-scale studies highlight that the procedure remains complex, with outcomes heavily influenced by tumor location and center experience [9]. For instance, lesions in the pancreatic head, including postpancreatectomy hemorrhage and bile leakage, are associated with increased morbidity. Furthermore, a critical and evolving challenge is the intraoperative management of the main pancreatic duct (MPD) [4,9]. A landmark 2025 prospective cohort study (CSPAC-MIEN-1) demonstrated that while MPD exposure, repair, or reconstruction (ERR) during MI-pEn significantly increased the incidence of perioperative complications such as clinically relevant pancreatic fistula (79.6% vs. 41.6%), it did not affect long-term metabolic function or quality of life [10]. This underscores a critical knowledge gap: the surgical community lacks a comprehensive understanding of how these advanced, high-risk scenarios impact the overall comparative outcomes of MI-pEn versus O-pEn [3,8].

The existing evidence is predominantly composed of small-sample, retrospective studies with significant heterogeneity and inherent biases [8]. High-level evidence, such as that provided by recent randomized controlled trials for other minimally invasive pancreatic procedures, is notably absent for enucleation, leaving a critical void in guiding evidence-based clinical decision-making. While previous systematic reviews have focused on this topic [8,11,12,13], they have been constrained by small sample sizes, limited inclusion of propensity score-matched (PSM) studies, and an inability to incorporate the most recent evidence on advanced minimally invasive techniques and their associated risks. Moreover, no prior meta-analysis has systematically compared robotic and laparoscopic approaches for pancreatic enucleation—a critical gap regarding the relative safety and efficacy of these two minimally invasive platforms.

Therefore, this systematic review and meta-analysis aims to integrate the most current and comprehensive data available to provide a robust evaluation of the short-term outcomes of various pancreatic enucleation procedures. For the first time, it directly compares robotic and laparoscopic approaches to clarify the safety, feasibility, and potential benefits of minimally invasive pancreatic enucleation (MI-pEn) in modern practice.

## 2. Materials and Methods

### 2.1. Literature Search Strategy

A computerized search of the PubMed, MEDLINE, Embase, and Web of Science databases was performed using the search terms “pancreas”, “pancreatic”, “minimally invasive surgery”, “laparoscopy”, “robotic”, “laproscopic”, “robot assisted”, “enucleation”, and “pancreatic enucleation”. The search period was from January 1990 to December 2025, without language restrictions.

This systematic review and meta-analysis were conducted in accordance with the PRISMA 2020 statement [14]. The completed PRISMA checklist is provided in the Supplementary Materials (Supplementary File S1). Ethical approval was not needed, since this study is a review of previously published studies. Our protocol was registered with PROSPERO, CRD420261332624.

### 2.2. Inclusion and Exclusion Criteria

The inclusion criteria were as follows: (1) study design: prospective or retrospective cohort studies, case–control studies, and randomized controlled trials; (2) patients undergoing pancreatic enucleation, with comparisons between laparoscopic and robot-assisted methods, and O-pEn group, comparing with laparoscopic or robot-assisted methods; (3) reporting of at least one short-term outcome measure; and (4) the article language was restricted to English.

The exclusion criteria were as follows: (1) nonoriginal research, such as reviews, expert opinions, conference abstracts, or case reports; (2) incomplete data or inability to extract data; (3) duplicate publications; (4) combined with other surgical methods; and (5) only pediatric patients.

### 2.3. Data Extraction and Quality Assessment

Two reviewers independently screened the literature and extracted data, including baseline characteristics (author, year, country, sample size, age, sex, BMI, tumor size, conversion, tumor location and distance from the main pancreatic duct); intraoperative outcomes (operation time, intraoperative blood loss, and R0 resection); and postoperative outcomes (pancreatic fistula, length of hospital stay, major morbidity (Clavien–Dindo grade ≥ III), wound infection and reoperation). Quality assessment was performed using the Newcastle-Ottawa Scale (NOS) [15]. The NOS includes three domains, selection of study groups (max 4 points), comparability of groups (max 2 points), and assessment of outcomes (max 3 points), with a total maximum score of 9. Studies scoring ≥ 6 points were considered high quality. Discrepancies in the assessment were resolved through discussion until a consensus was reached.

### 2.4. Outcome Measures and Definition

Primary outcomes included clinically relevant POPF (ISGPF grade B/C). Secondary outcomes included major morbidity (Clavien–Dindo ≥ III), operation time, intraoperative blood loss, hospital stay, R0 resection, wound infection, and reoperation. Clinically relevant pancreatic fistula was defined according to the International Study Group on Pancreatic Fistula (ISGPF) 2005 and 2016 criteria as a drain fluid amylase level greater than three times the upper limit of the normal serum amylase level, associated with corresponding clinical symptoms or requiring clinical intervention (Grade B/C) [16,17]. Secondary outcomes included severe postoperative complications categorized as Clavien–Dindo grade ≥ III, which referred to complications requiring endoscopic, interventional, or surgical management under general anesthesia, or those that were life-threatening or resulted in single- or multi-organ dysfunction [18]. The operation time was measured in minutes from skin incision to wound closure. Intraoperative blood loss was estimated in millilitres on the basis of anesthetic records or surgical reports. The length of hospital stay was defined as the time from the day of surgery to the day of discharge that met standard clinical criteria, including tolerance of oral intake, adequate pain control, and the absence of unresolved complications [19]. R0 resection was defined as the absence of tumor cells at the surgical margins on microscopic examination [20], and wound infection was defined according to the Centers for Disease Control and Prevention (CDC) criteria or as reported in individual studies typically involving purulent drainage or positive culture from the surgical site [21]. Reoperation was defined as any unplanned surgical intervention occurring during the same hospitalization or within 30 days following discharge [22].

### 2.5. Statistical Analysis

Statistical analyses were performed using RevMan 5.4.1 software and R 4.3.0. Odds ratios (ORs) were used as effect measures for categorical variables, and mean differences (MDs) were used for continuous variables, all of which are reported with 95% confidence intervals (CIs). For studies reporting continuous variables as medians and ranges, we converted these to means and standard deviations using the validated method described by Wan et al. (2014) [23]. Heterogeneity among the included studies was assessed using Cochran’s Q test and *I*^2^ statistics. Significant heterogeneity (*p* < 0.1 and *I*^2^ > 50%) led to the use of a random effects model; otherwise, a fixed effects model was applied. A subgroup analysis was performed to explore potential sources of heterogeneity. A sensitivity analysis was performed by sequentially excluding each study to test the stability of the pooled results. Publication bias was assessed using funnel plots and Egger’s test. All the statistical analyses were two-sided, and *p* < 0.05 was considered to indicate statistical significance.

## 3. Results

### 3.1. Study Selection

**Figure 1:**
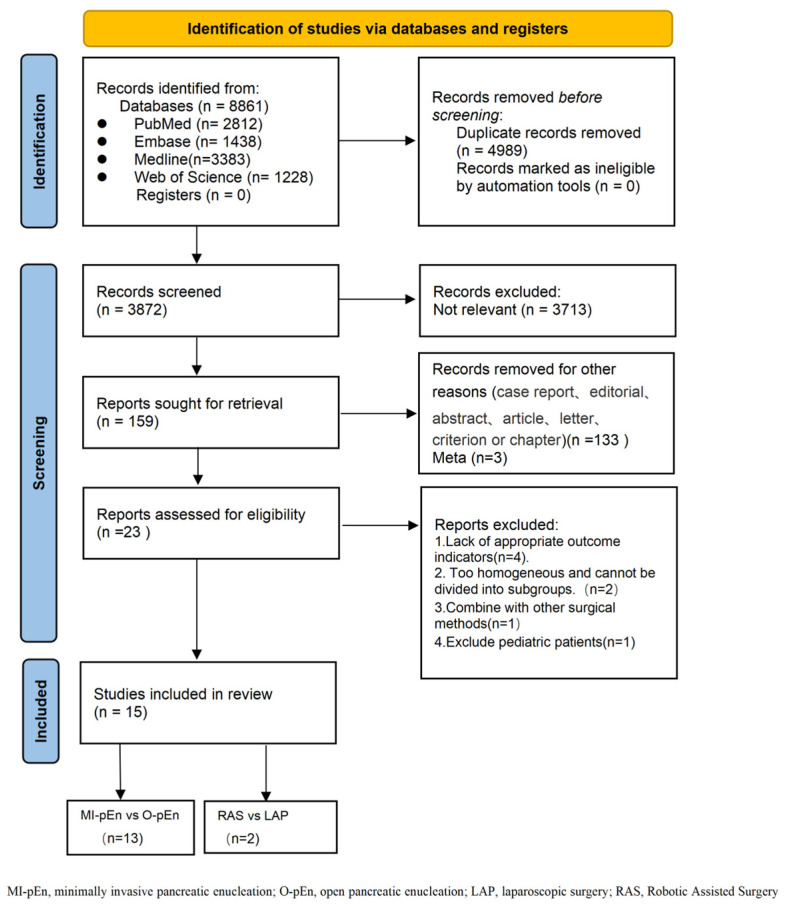
PRISMA flow diagram for the systematic review.

The initial lite underwent title and abstract screening, of which 3215 were excluded because they were irrelevant. The remaining search yielded 4148 records. After removing duplicates (*n* = 784), 3364 articles were retrieved for full-text review. Following the exclusion of case reports, editorials, conference abstracts, meta-analyses (*n* = 3) [8,12,13], studies lacking appropriate outcome indicators (*n* = 4) [24,25,26,27], studies with homogenous populations that could not be subgrouped (*n* = 2) [28,29], studies combining other surgical procedures (*n* = 1) [30], and studies including pediatric patients (*n* = 1) [31], a total of 15 studies met the inclusion criteria for this systematic review. The literature screening process is detailed in the PRISMA flow diagram (Figure 1). Among these, 13 were comparative studies of minimally invasive versus open enucleation (463 patients in the MI-pEn group and 547 patients in the O-pEn group). Two studies [32,33] compared robotic and laparoscopic enucleation. The included studies were published between 2014 and 2025 and were retrospective or prospective cohort studies. Most studies scored ≥6 on the NOS, indicating moderate to high quality.

### 3.2. Baseline Characteristics and Quality Assessment

The included studies were published between 2014 and 2025. All were retrospective cohort studies, with three utilizing propensity score matching. The Newcastle-Ottawa Scale (NOS) scores ranged from 5 to 8, with 11 studies (73.3%) considered high quality (NOS ≥ 6) [6,32,33,34,35,36,37,38,39,40,41]. The NOS scores of all included studies are shown in Appendix A. The MI-pEn and O-pEn groups were comparable in terms of age, sex distribution, body mass index (BMI), tumor size, and tumor location, and the majority of tumors (63%) were located in the body or tail of the pancreas. The basic characteristics of the included studies are shown in Table 1.

### 3.3. The Primary Outcome

**Figure 2:**
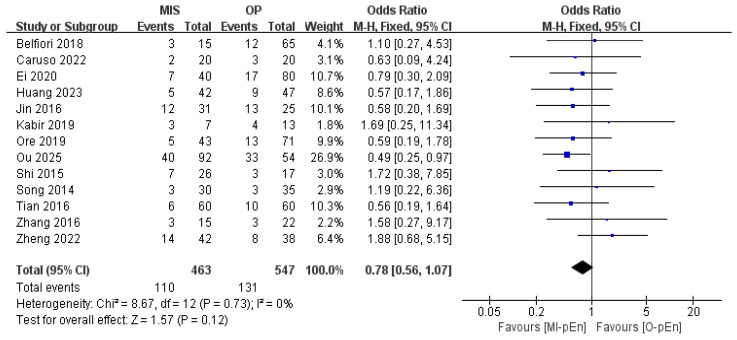
The forest diagram displays the primary outcome—clinically relevant POPF (ISGPF Grade B/C) [7,34,35,36,37,38,39,40,41,42,43,44,45]. Each blue square represents an individual study; the square size reflects the study’s weight. Horizontal lines show 95% confidence intervals (CIs). The vertical line at OR = 1 indicates no difference between MI-pEn and O-pEn. The diamond represents the pooled odds ratio (OR) using a fixed-effects model (*I*^2^ = 0%); an OR < 1 favors MI-pEn. Blue colour indicates that the effect measure is OR.

**Figure 3:**
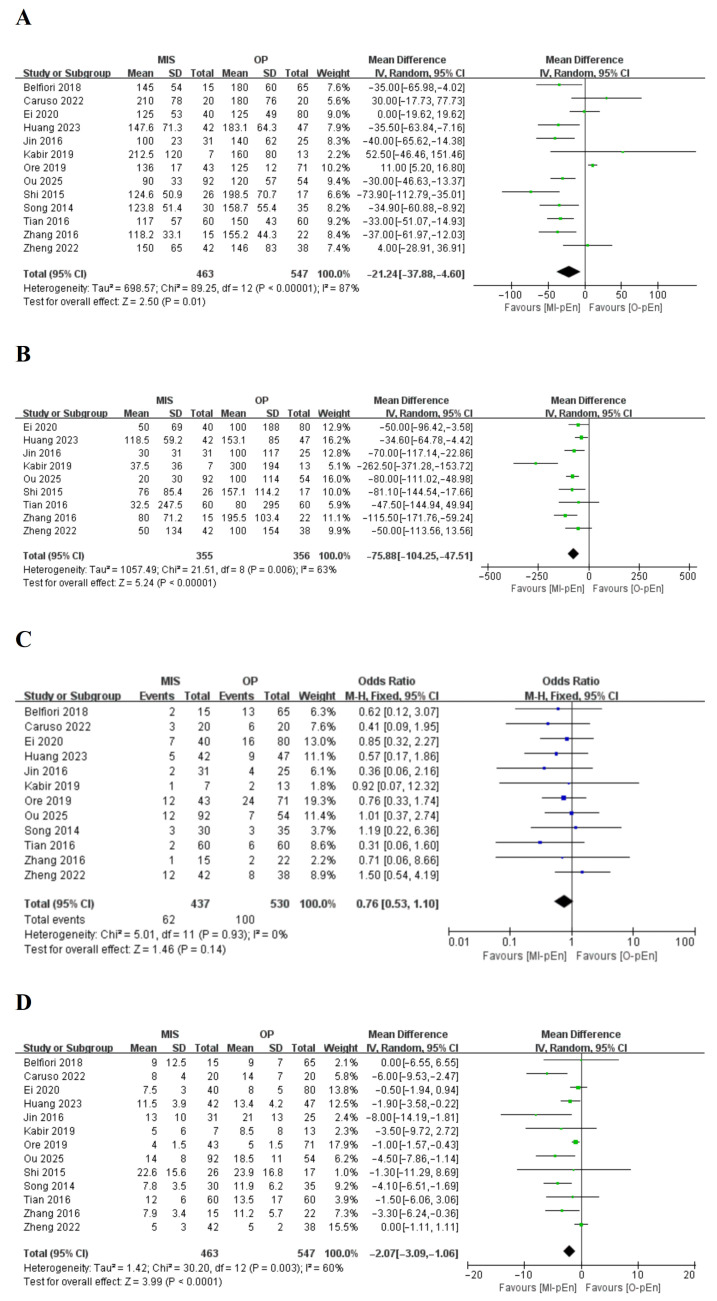

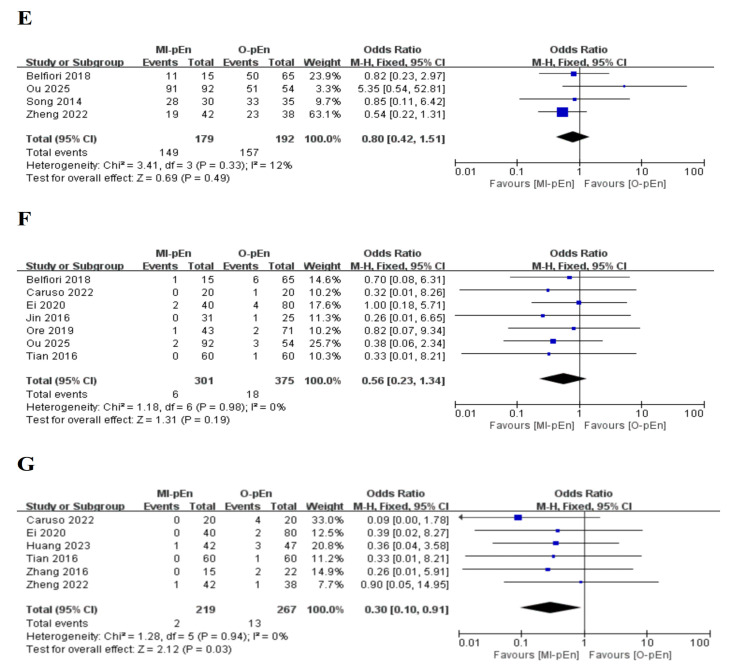
Forest plots of secondary outcomes. [7,34,35,36,37,38,39,40,41,42,43,44,45] (**A**) Operation time (minutes); (**B**) intraoperative blood loss (mL); (**C**) major morbidity (Clavien–Dindo grade ≥ III); (**D**) length of hospital stay (days); (**E**) R0 resection; (**F**) reoperation; (**G**) wound infection. For each outcome, the effect measure is either the mean difference (MD) for continuous variables (**A**,**B**,**D**) or the odds ratio (OR) for categorical variables (**C**,**E**,**F**,**G**). In each forest plot, the left side favors MI-pEn (MD < 0 or OR < 1), and the right side favors O-pEn (MD > 0 or OR > 1). Green squares represent continuous outcomes (MD: operation time, blood loss, hospital stay); blue squares represent dichotomous outcomes (OR: major morbidity, R0 resection, reoperation, wound infection). Square size reflects study weight; horizontal lines show 95% CIs. The vertical line marks no effect (MD = 0 or OR = 1). The diamond denotes the pooled effect estimate with its 95% CI, using a fixed- or random-effects model depending on heterogeneity (*I*^2^ values in main text and Table 2). Heterogeneity was substantial for operation time (*I*^2^ = 87%), moderate for blood loss (*I*^2^ = 63%) and hospital stay (*I*^2^ = 60%), and low or zero for other outcomes. Study-specific sample sizes are given as MI-pEn/O-pEn.

The results of the meta-analysis revealed that the primary outcome, CR-POPF (ISGPF grade B/C), was not significantly different between the CR-POPF rates of MI-pEn and O-pEn (OR = 0.78; 95% CI: 0.56–1.07; *p* = 0.12), with no heterogeneity among the studies (*I*^2^ = 0%). In all the forest plots (Figure 2 and Figure 3), an effect estimate to the left of the vertical line indicates a potential advantage for MI-pEn, whereas an estimate to the right favors O-pEn. The primary outcomes are shown in Figure 2 and Table 2.

### 3.4. Secondary Outcomes

With respect to the secondary outcomes, the direction of the effect consistently increased the MI-pEn when significant differences were observed (i.e., shorter operation time, less blood loss, shorter hospital stay, and lower wound infection rate), as shown in Figure 3 and summarized in Table 2. Compared with O-pEn, MI-pEn resulted in significantly better perioperative outcomes: the mean operation time was 21.24 min (95% CI: 37.88–4.60; *p* = 0.01), the mean intraoperative blood loss was 75.88 mL (95% CI: −104.25–47.51; *p* < 0.00001), the mean length of hospital stay was 2.07 days (95% CI: −3.09–1.06; *p* = 0.001), and the mean degree of wound infection was 0.30 (95% CI: 0.1–0.91; *p* = 0.03). Heterogeneity was substantial for operation time (*I*^2^ = 87%) and moderate for blood loss (*I*^2^ = 63%) and hospital stay (*I*^2^ = 60%). There were no significant differences between the two approaches in terms of the rates of major morbidity (OR = 0.76; 95% CI: 0.53–1.10; *p* = 0.14), R0 resection (OR = 0.80; 95% CI: 0.42–1.51; *p* = 0.49), or reoperation (OR = 0.56; 95% CI: 0.23–1.34; *p* = 0.19). The conversion rate from minimally invasive surgery to open surgery was 4.3% (20/463). In direct comparisons between robotic and laparoscopic enucleation, no significant differences were observed in any of the evaluated outcomes, including CR-POPF (OR = 0.62, 95% CI: 0.22–1.78, *p* = 0.38), operation time (MD = 23.94, 95% CI: –21.47 to 69.35, *p* = 0.30), intraoperative blood loss (MD = 10.63, 95% CI: –15.80 to 37.06, *p* = 0.43), or length of hospital stay (MD = 1.45, 95% CI: –0.72 to 3.63, *p* = 0.19).

### 3.5. Subgroup Analysis

The subgroup analyses were performed according to the following aspects: surgical approach (robotic vs. open, laparoscopic vs. open, and laparoscopic vs. robotic); tumor location (pancreatic head and neck); publication year (2020 or later); sample size (greater than 40); and study quality (NOS score ≥ 6). The results of the subgroup analysis of the outcomes are shown in Table 3 and Figure 3. Subgroup analyses revealed that the benefit of MI-pEn on CR-POPF was particularly evident in robot-assisted procedures: compared with O-pEn, robotic enucleation was associated with a significantly lower CR-POPF rate (OR = 0.59, 95% CI: 0.37–0.95, *p* = 0.03; *I*^2^ = 0%). In the comparisons between the laparoscopic and open groups, no difference in the CR-POPF was observed (OR = 1.36; 95% CI: 0.40–4.58; *p* = 0.62; *I*^2^ = 0%). Direct comparisons between laparoscopic and robotic enucleation revealed no statistically significant differences in any outcome, including CR-POPF (OR = 0.62; 95% CI: 0.22–1.77; *p* = 0.37; *I*^2^ = 0%). In other predefined subgroups—studies with a NOS ≥ 6, tumors located in the pancreatic head/neck, and studies with sample sizes ≥ 40—the point estimates for CR-POPF consistently favored MI-pEn (ORs ranging from 0.62 to 0.73), although the confidence intervals crossed unity, and heterogeneity remained low (*I*^2^ = 0–18%). Sensitivity analysis by sequential omission of each study confirmed the robustness of the pooled estimates, and funnel plot inspection along with Egger’s test indicated no significant publication bias for the primary outcomes.

### 3.6. Sensitivity Analysis and Publication Bias

**Figure 4:**
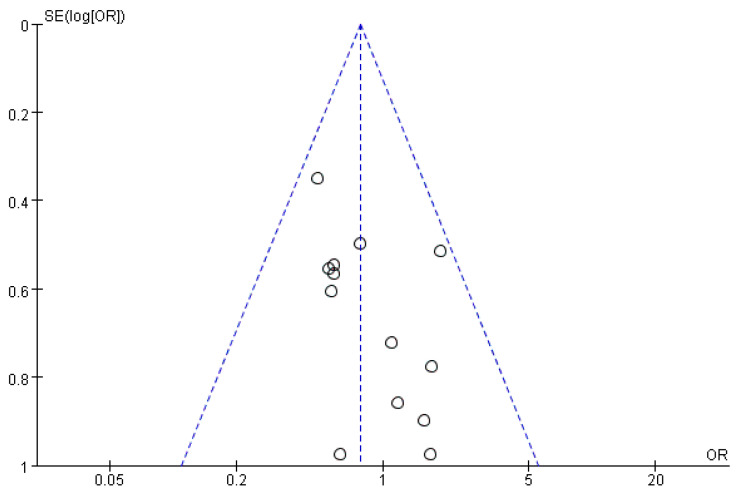
Funnel plots for clinically relevant POPF. In this funnel plot, each circle represents an individual study included in the meta-analysis of clinically relevant postoperative pancreatic fistula (CR-POPF). The vertical dashed line indicates the pooled effect estimate (log odds ratio). The two diagonal dashed lines (the pseudo-95% confidence interval funnel) represent the expected region of symmetry under the assumption of no publication bias; studies are expected to be distributed symmetrically within this funnel. Symmetry of the circles around the vertical line suggests absence of publication bias, whereas asymmetry would indicate potential bias or small-study effects.

Sensitivity analysis was performed by sequentially excluding each study, and the results revealed that no single study significantly altered the pooled effect size, indicating that the results of the meta-analysis were stable. Publication bias was assessed using both Begg’s and Egger’s tests. Funnel plots for primary outcomes revealed symmetry, and Egger’s test revealed no significant publication bias (*p* > 0.05), as shown in Figure 4.

## 4. Discussion

This systematic review and meta-analysis demonstrated that minimally invasive pancreatic enucleation (MI-pEn) is a safe and effective alternative to open enucleation (O-pEn), offering a shorter operative time, less blood loss, a shorter hospital stay, and fewer wound infections, while maintaining comparable rates of CR-POPF, major morbidity, R0 resection, and reoperation. Direct comparisons between the robotic and laparoscopic approaches revealed no significant differences in any short-term outcomes. However, in the subgroup analysis of open surgery, robotic—but not laparoscopic—enucleation was associated with a significantly lower rate of pancreatic fistula.

Compared with O-pEn, MI-pEn was associated with a 21-min reduction in operation time, a decrease in blood loss of 75.88 mL, and a 2-day shorter hospital stay. These benefits align with the established advantages of minimally invasive surgery across various surgical disciplines, including enhanced visualization, reduced tissue trauma, and accelerated postoperative recovery [3,8]. Importantly, these improvements were achieved without compromising surgical safety, as evidenced by the comparable rates of major morbidity (Clavien–Dindo grade ≥ III) and CR-POPF between the two approaches [7].

Notably, the overall CR-POPF rate in the MI-pEn group did not differ significantly from that in the O-pEn group (OR = 0.78; 95% CI: 0.56–1.07; *p* = 0.12). However, subgroup analysis based on surgical platform revealed a significant reduction in the CR-POPF associated with robotic-assisted enucleation compared with that associated with open surgery (OR = 0.59; 95% CI: 0.37–0.95; *p* = 0.03), whereas no such difference was observed for laparoscopic enucleation (OR = 1.36; 95% CI: 0.40–4.58; *p* = 0.62). These findings suggest that the enhanced three-dimensional visualization, tremor filtration, and articulated instrumentation of robotic platforms may facilitate more precise dissection near the main pancreatic duct, thereby reducing the risk of pancreatic fistula [39,44,46]. Direct comparisons between laparoscopic and robotic enucleation, although limited by small sample sizes, revealed no statistically significant differences in any outcome, indicating that both minimally invasive approaches may offer comparable short-term efficacy [33,47,48]. These findings should be interpreted as hypothesis-generating only, given the limited number of studies and small sample sizes in the robotic subgroup. Direct comparative studies with larger cohorts are needed to confirm a potential fistula-reduction benefit.

Heterogeneity across studies was substantial for operative time, blood loss, and hospital stay, likely reflecting variations in surgical technique, center experience, patient selection, and study design. The observed heterogeneity likely reflects differences in tumor location (head vs. body/tail), surgeon experience, conversion rates, and institutional protocols. For instance, studies predominantly including head tumors reported longer operative times and greater blood loss, contributing to the overall *I*^2^ values. Subgroup and sensitivity analyses confirmed the robustness of the pooled estimates, with consistent effect directions across studies of higher quality (NOS ≥ 6), those focusing on head/neck tumors, and those published since 2020. The funnel plot symmetry and nonsignificant Egger’s test results suggested a low likelihood of publication bias for primary outcomes [49]. Beyond statistical heterogeneity, clinical heterogeneity in patient selection, surgical technique, and perioperative care may also influence outcomes.

The conversion rate from minimally invasive to open surgery was 4.3%, which is acceptably low and comparable to the rates reported for other minimally invasive pancreatic procedures. These findings support the feasibility of MI-pEn in appropriately selected patients when performed at experienced centers.

Despite the strengths of this meta-analysis, including its comprehensive literature search, rigorous methodology, and inclusion of recent high-quality studies, several limitations must be acknowledged. First, all included studies were observational (retrospective or prospective cohort designs), which are inherently susceptible to selection bias and confounding, despite the use of propensity score matching in some studies. The absence of prospective studies and the narrowing of the publication timeline reflect the limited high-quality evidence available, which may introduce selection and publication bias. Second, incomplete reporting of baseline characteristics, such as tumor distance from the main pancreatic duct and intraoperative ultrasound use, precluded more detailed subgroup analyses and may have influenced outcome heterogeneity. In particular, the distance from the main pancreatic duct and tumor location—key determinants of surgical complexity—have been inconsistently reported across studies. This incomplete information may have introduced unmeasured confounding, potentially affecting the pooled estimates and the interpretation of outcomes. Third, the number of studies available for certain comparisons, particularly direct laparoscopic versus robotic comparisons, was limited, reducing statistical power and precision. Fourth, the lack of standardized reporting for some outcomes across studies may have introduced measurement bias. Definitions of wound infection varied across the included studies, with some applying CDC criteria and others using clinical assessments such as purulent drainage or positive culture. This lack of harmonization may have introduced measurement bias and contributed to heterogeneity. Therefore, the discharge time indeed varies across different countries, and is indeed a source of heterogeneity. In contrast, hospital stay was uniformly defined as the time from surgery to discharge on the basis of standard clinical criteria, minimizing definition-related variability for this outcome. Importantly, this analysis is limited to short-term outcomes. Long-term functional outcomes (exocrine/endocrine insufficiency), recurrence rates, and quality of life, which only a small number of studies have been reported, were not assessed and should be prioritized in future research.

Looking forward, the integration of advanced technologies and data-driven approaches holds promise for further improving outcomes in pancreatic enucleation. Artificial intelligence could aid in preoperative risk stratification and POPF prediction, while intraoperative image-guided navigation (e.g., augmented reality, fluorescence imaging) may improve real-time identification of tumor margins and the main pancreatic duct. Machine learning models that integrate perioperative variables could further personalize surgical decision-making. These tools require prospective validation but hold promise for optimizing outcomes in minimally invasive enucleation.

Future research should prioritize well-designed, multicenter prospective cohort studies or randomized controlled trials with standardized outcome definitions and longer follow-up periods [33]. Such studies should also investigate the impact of adjunctive technologies, such as intraoperative ultrasound and pancreatic duct stenting, as well as the influence of surgeon experience and learning curves on outcomes [6,50]. Additionally, health economic evaluations comparing laparoscopic and robotic enucleation are needed to inform resource allocation and guideline development.

In summary, compared with open surgery, MI-pEn offers significant perioperative benefits with comparable safety. Robotic and laparoscopic approaches appear equally effective, although robotic surgery may confer an additional advantage in terms of fistula prevention that requires further study.

## 5. Conclusions

This meta-analysis confirms that minimally invasive pancreatic enucleation (MI-pEn) is safe and feasible and offers a shorter operative time, less blood loss, and a shorter hospital stay than open surgery does, without increasing the incidence of pancreatic fistula, major morbidity, or reoperation. Subgroup analysis revealed that compared with open surgery, robotic-assisted enucleation, but not laparoscopic enucleation, was associated with a significantly lower pancreatic fistula rate. However, direct robotic–laparoscopic comparisons revealed no significant differences in any short-term outcomes, suggesting comparable safety and efficacy. The potential benefit of robotics in fistula reduction warrants further validation through high-quality studies with long-term follow-up, including assessments of oncological and functional outcomes. However, these findings require validation in larger prospective studies.

## Figures and Tables

**Table 1:** Basic Characteristics of Included Studies.

Author	Year	Country	Study Design	Group	No of Patient	Age	Gender (M/F)	BMI	Tumor Size (cm)	Tumor Location (H-B/T)	MPD Distance from Tumor (mm)	Conversion (%)
Belfiori et al. [42]	2018	Germany	Retrospective Cohort	MI-pEn	15	39 ± 15	8/7	27 ± 9	1.25 ± 0.5	4/11	N	3
				O-pEn	65	48.5 ± 15	18/47	24.3 ± 5	1.4 ± 0.6	37/22		/
Caruso et al. [43]	2022	Spain	Case-Matched Study	MI-pEn	20	61 ± 12.5	12/8	24 ± 2.8	1.8 ± 0.4	6/14	N	1
				O-pEn	20	67.78 ± 7.8	15/5	20 ± 2.6	2 ± 0.3	5/15		/
Ei et al. [34]	2020	Germany	Retrospective Cohort (Matched)	MI-pEn	40	55 ± 5	13/27	24.9 ±1.5	2.2 ± 0.3	9/31	1.8 (1–3.2)	3
				O-pEn	80	58 ± 3	29/51	25.3 ± 1.6	2.3 ± 0.3	24/56	2.1 (1–3.3)	/
Huang et al. [35]	2023	China	Retrospective Cohort	MI-pEn	42	49.1 ± 14.5	15/27	23.2 ± 3.8	3.29 ± 1.30	17/25	N	N
				O-pEn	47	48.0 ± 11.7	21/26	24.0 ± 3.2	3.42 ± 1.59	26/21		/
Jin et al. [36]	2016	China	Retrospective Cohort	MI-pEn	31	51.0 ± 5	11/20	23.8 ± 3.1	2.0 ± 0.2	16/15	5 (5–5.8)	0
				O-pEn	25	51.0 ± 5	12/13	23.6 ± 4.3	2.5 ± 0.3	19/6	4.8 (2.4–5)	/
Kabir et al. [7]	2019	Singapore	Retrospective Cohort (PS-adjusted)	MI-pEn	7	43 ± 13	2/5	22.3 ±5.8	1.5 ± 0.1	3/4	N	0
				O-pEn	13	56 ± 8	6/7	28.2 ±1.8	1.45 ± 0.2	9/4		/
Ore et al. [44]	2019	American	Retrospective Cohort (NSQIP)	MI-pEn	43	56 ± 4	30/13	30 ± 1.8	3 ± 0.4	N	N	7
				O-pEn	71	60 ± 5	39/32	28 ± 2.3	3 ± 0.4			/
Ou et al. [37]	2025	UK	Retrospective Cohort	MI-pEn	92	49.7 (15.4)	39/53	24.02 ± 1.1	2.0 ± 0.2	All in head	3.1 (1.7~5.1)	0
				O-pEn	54	49.5 (14.6)	24/30	25.0 ± 1.3	2.3 ± 0.4		2.0 (0.5~3.9)	/
Shi et al. [45]	2015	China	Retrospective Cohort	MI-pEn	26	50.1 ± 14.1	10/16	23.1 ± 5.6	2.3 ± 1.2	10/16	1–2	0
				O-pEn	17	54.6 ± 17.2	6/11	22.4 ± 4.1	3.5 ± 1.9	9/8		/
Song et al. [38]	2014	Korea	Retrospective Cohort	MI-pEn	30	51.2 ± 11.8	17/48	23.3 ± 3.9	2.8 ± 1.6	31/34	2–3	N
				O-pEn	35	51.3 ± 13.3		24.5 ± 3.9	3.1 ± 1.9			/
Tian et al. [39]	2016	China	Retrospective Cohort (PS-matched)	MI-pEn	60	45.2 (13.1)	21/39	25.9 ± 3	13.7 (3.4)	27/33	>2	3
				O-pEn	60	43.0 (12.9)	17/43	25.0 ± 6.4	13.8 (3.6)	30/30		/
Zhang et al. [41]	2016	China	Retrospective Cohort	MI-pEn	15	49.5 ± 13.1	6/9	23.4 ± 3.2	3.4 ± 1.8	6/9	N	1
				O-pEn	22	48.4 ± 13.5	5/17	22.5 ± 3.9	3.3 ± 2.7	15/7		/
Zheng et al. [40]	2022	American	Retrospective Cohort (PS-matched)	MI-pEn	42	57.5 ± 14.8	24/18	N	1.50 ± 0.2	15/27	N	2
				O-pEn	38	56.3 ± 15.3	23/15		1.50 ± 0.1	27/11		/
Najafi et al. [32]	2020	Germany	Retrospective Cohort	Lap	8	51 ± 15	9/10	28 ± 4.3	17 ± 15	1/18	N	0
				RAS	11							/
Yin et al. [33]	2024	China	Retrospective Cohort	Lap	26	43.2 ± 9.4	6/20	28.8 ± 4.5	1.7 ± 0.1	16/10	N	5
				RAS	33	46.3 ± 11.8	11/22	26.9 ± 3.2	1.5 ± 0.1	16/17		/

N, not available; M/F, male/female; tumor location (H-B/T): H = head and neck of the pancreas; B/T = body and tail of the pancreas; MPD distance (mm): distance from the main pancreatic duct; Conversion (%): conversion rate to laparotomy; MI-pEn, minimally invasive pancreatic enucleation; O-pEn, open pancreatic enucleation; Lap, laparoscopic surgery; RAS, robotic-assisted surgery.

**Table 2:** Results of the Meta-analysis.

Outcomes	No of Patients	Relative Effect (MD/OR)	95% CI	*p*	*I* ^2^
Pancreatic Fistula (B/C)	1010	0.78	0.56, 1.07	0.12	0%
Operation time (min)	1010	−21.24	−37.88, −4.60	0.01	87%
Blood loss (mL)	711	−75.88	−104.25, −47.51	0.00001	63%
LOS (days)	1010	−2.07	−3.09, −1.06	0.001	60%
Major morbidity (≥III)	967	0.76	0.53, 1.10	0.14	0%
R0 resection	371	0.8	0.42, 1.51	0.49	12%
Reoperation	676	0.56	0.23, 1.34	0.19	0%
Wound infection	468	0.30	0.10, 0.91	0.03	0%

LOS, length of hospital stay; OR, odds ratio; MD, mean difference; CI, confidence interval. For continuous outcomes, a negative MD favors MI-pEn. For categorical outcomes, an OR < 1 favors MI-pEn.

**Table 3:** Subgroup Analysis of the Outcomes.

Outcomes	No. of Patients	Relative Effect (MD/OR)	95% CI	*p*	*I* ^2^
Robot vs. open					
Operation time (min)	405	−32.54	−51.98, −13.10	0.001	65%
Blood loss (mL)	356	−75.85	−99.14, −52.56	0.00001	0%
LOS (days)	405	−4.64	−6.63, −2.65	0.00001	0%
Pancreatic Fistula (B/C)	405	0.59	0.37, 0.95	0.03	0%
Major morbodity (≥3 grade)	362	0.58	0.30, 1.12	0.11	0%
Lap vs. open					
Operation time (min)	102	−35.99	−53.99, −17.99	0.0001	0%
LOS (days)	102	−3.78	−5.64, −1.92	0.0001	0%
Pancreatic Fistula (B/C)	102	1.36	0.40, 4.58	0.62	0%
Major morbodity (≥3 grade)	102	1.01	0.25, 4.01	0.99	0%
Lap vs. RAS					
Operation time (min)	78	23.94	−21.47, 69.35	0.3	60%
Blood loss (mL)	78	10.63	−15.80, 37.06	0.43	12%
LOS (days)	78	1.45	−0.72, 3.63	0.19	8%
Pancreatic Fistula (B/C)	78	0.62	0.22, 1.77	0.37	0%
Major morbodity (≥3 grade)	78	0.92	0.26, 3.27	0.90	0%
≥6 ^c^					
Operation time (min)	827	−21.14	−39.09, −3.20	0.02	88%
Blood loss (mL)	648	−61.75	−78.15, −45.35	<0.00001	30%
LOS (days)	827	−1.78	−2.8, −0.76	0.0006	64%
Pancreatic Fistula (B/C)	827	0.71	0.51, 1.01	0.06	0%
Major morbodity (≥3 grade)	827	1.01	0.25, 4.01	0.99	0%
R0 resection	291	0.79	0.38, 1.65	0.53	41%
Wound infection	446	0.40	0.12, 1.39	0.15	0%
Reoperation	556	0.56	0.21, 1.52	0.25	0%
tumor located in the pancreatic head and pancreatic neck					
Operation time (min)	238	−33.94	−48.18, −19.71	<0.00001	0%
Blood loss (mL)	238	−78.44	−102.04, −54.84	<0.00001	49%
LOS (days)	238	−3.13	−5.1, −1.16	0.002	0%
Pancreatic Fistula (B/C)	238	0.62	0.36, 1.08	0.09	0%
Major morbodity (≥3 grade)	195	0.88	0.38, 2.03	0.76	0%
Reoperation	160	0.47	0.09, 2.39	0.36	0%
≥2020 ^a^					
Operation time (min)	475	−10.78	−31.11,9.54	0.3	67%
Blood loss (mL)	453	−55.01	−73.75, −36.28	<0.00001	31%
LOS (days)	475	−1.96	−3.67, −0.24	0.03	76%
Pancreatic Fistula (B/C)	475	0.73	0.47, 1.12	0.15	18%
Major morbodity (≥3 grade)	475	0.87	0.54, 1.42	0.58	0%
R0 resection	226	1.32	0.15, 11.99	0.8	70%
Wound infection	329	0.30	0.08, 1.10	0.07	0%
Reoperation	306	0.56	0.18, 1.68	0.3	0%
≥40 ^b^					
Operation time (min)	749	−16.18	−35.63, 3.27	0.1	88%
Blood loss (mL)	555	−54.74	−73.14, −36.35	<0.00001	8%
LOS (days)	749	−0.91	−1.36, −0.46	<0.0001	32%
Pancreatic Fistula (B/C)	749	0.71	0.49, 1.03	0.07	0%
Major morbodity (≥3 grade)	749	0.81	0.54, 1.21	0.30	0%
R0 resection	306	0.79	0.4, 1.55	0.5	41%
Wound infection	409	0.44	0.11, 1.71	0.24	0%
Reoperation	580	0.63	0.24, 1.64	0.30	0%

LOS, length of hospital stay; OR, odds ratio; MD, mean difference; CI, confidence interval; ^a^ Studies published in the year 2020 or later; ^b^ Studies with a sample size greater than 40; ^c^ Studies with NOS scores ≥ 6. Lap, laparoscopic surgery; Open, open surgery; RAS, Robotic-Assisted Surgery.

## Data Availability

No new data were generated or analyzed in this study.

## Supplementary Materials

The following supporting information can be downloaded at: https://www.mdpi.com/article/10.3390/jcm15093543/s1, File S1: PRISMA 2020 Checklist.

## Appendix A

**Table A1:** Assessment of Literature Quality.

	Selection ^1^	Comparability ^2^	Assessment of Outcome ^3^	Total Points
Belfiori et al. [41]	★★★★	/	★	5
Caruso et al. [42]	★★★	★★	/	5
Ei et al. [33]	★★★★	★★	/	6
Huang et al. [34]	★★★★	★	★★	7
Jin et al. [35]	★★★★	/	★★★	7
Kabir et al. [7]	★★★	★★	/	5
Ore et al. [43]	★★★★	★★	★	7
Ou et al. [36]	★★★★	★	★★	7
Shi et al. [44]	★★★★	/	★	5
Song et al. [37]	★★★★	/	★★	6
Tian et al. [38]	★★★★	★★	/	6
Zhang et al. [40]	★★★★	★	★★	7
Zheng et al. [39]	★★★★	★★	★★	8
Najafi et al. [31]	★★★★	/	★★	6
Yin et al. [32]	★★★★	★	★★	7

^1^ Selection of study groups (max 4 points); ^2^ comparability of groups (max 2 points); ^3^ assessment of outcome (max 3 points); ★ represents 1 point.
